# Mesenchymal tumor cells drive adaptive resistance of *Trp53^−/−^* breast tumor cells to inactivated mutant *Kras*

**DOI:** 10.1002/1878-0261.13220

**Published:** 2022-04-23

**Authors:** Linda J. van Weele, Sabra I. Djomehri, Shang Cai, Jane Antony, Shaheen S. Sikandar, Dalong Qian, William H. D. Ho, Robert B. West, Ferenc A. Scheeren, Michael F. Clarke

**Affiliations:** ^1^ Institute for Stem Cell Biology and Regenerative Medicine School of Medicine Stanford University CA USA; ^2^ Department of Stem Cell Biotechnology California State University Channel Islands Camarillo CA USA; ^3^ Department Pathology Stanford University Medical Center Palo Alto CA USA; ^4^ 4501 Department of Medical Oncology Leiden University Medical Center The Netherlands; ^5^ Present address: 557712 Westlake University Hangzhou China; ^6^ Present address: Department of Molecular, Cell and Developmental Biology University of California Santa Cruz CA USA

**Keywords:** breast cancer, Kras, mouse model, treatment adaptation

## Abstract

As precision medicine increases the response rate of treatment, tumors frequently bypass inhibition, and reoccur. In order for treatment to be effective long term, the mechanisms enabling treatment adaptation need to be understood. Here, we report a mouse model that, in the absence of p53 and the presence of oncogenic *Kras^G12D^*, develops breast tumors. Upon inactivation of *Kras^G12D^*, tumors initially regress and enter remission. Subsequently, the majority of tumors adapt to the withdrawal of *Kras^G12D^* expression and return. *Kras^G12D^*‐independent tumor cells show a strong mesenchymal profile with active RAS‐RAF‐MEK‐ERK (MAPK/ERK) signaling. Both *Kras^G12D^*‐dependent and *Kras^G12D^*‐independent tumors display a high level of genomic instability, and *Kras^G12D^*‐independent tumors harbor numerous amplified genes that can activate the MAPK/ERK signaling pathway. Our study identifies both epithelial‐mesenchymal transition (EMT) and active MAPK/ERK signaling in tumors that adapt to oncogenic *Kras^G12D^* withdrawal in a novel *Trp53^−/−^* breast cancer mouse model. To achieve long‐lasting responses in the clinic to RAS‐fueled cancer, treatment will need to focus in parallel on obstructing tumors from adapting to oncogene inhibition.

## Graphical abstract

We report on a novel *Trp53^−/−^* Kras^G12D^‐fueled breast cancer mouse model. Primary tumors regress upon Kras^G12D^ inactivation, followed by Kras^G12D^‐independent reactivated growth. Reactivated tumors are mesenchymal. Both primary and reactivated tumors display genomic instability and active MAPK/ERK signaling. Activate signaling in reactivated tumors could possibly be explained by the increase in the amplification of genes that can activate MAPK/ERK signaling.

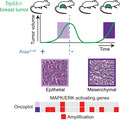

**Abbreviations**
CNA: copy number alteration
Cre: Cre recombinase
dox: doxycycline
EMT: epithelial‐mesenchymal transition
GSEA: gene set enrichment analysis
KO: knockout
pERK1/2: phosphorylated ERK1/2
PT: primary tumor
RT: reactivated tumor
RTK: receptor tyrosine kinase
SNV: single nucleotide variant
ssGSEA: single sample GSEA
TNBC: triple‐negative breast cancer
WES: whole exome sequencing

## 1 Introduction

Both tumor suppressor TP53 and oncogene Kras are commonly altered in cancer and are known drivers of breast cancer. While *TP53* is the most commonly mutated gene in breast cancer, Kras is more frequently mutated in other cancer types [1]. Nevertheless, introducing the expression of mutant *Kras^G12D^* in normal human mammary epithelium is sufficient to induce tumor formation [2]. Furthermore, the RAS‐RAF‐MEK‐ERK (hereafter referred to as MAPK/ERK) signaling pathway is frequently overactivated in all types of breast cancer, particularly in the molecular subtype basal‐like breast cancer. In basal‐like breast cancer, amplifications of MAPK/ERK pathway genes are common and *Kras* amplifications are detected in 32% of tumors [3, 4]. Basal‐like breast cancer frequently overlaps with a triple‐negative breast cancer (TNBC) diagnosis. Due to the lack of targeted or hormonal therapy for TNBC patients, the TNBC subtype has the worst prognosis with a 4‐year relative survival of 77.0% [5].

To study the effect of oncogenic *Kras* expression in breast cancer *in vivo*, we chose to use a *Kras^G12D^*‐inducible mouse model. The expression of *Kras^G12D^* mimics the overactivation of the MAPK/ERK pathway, as the continuously active Kras^G12D^ is the initial driver of this pathway. In addition, we aimed to shed light on what mechanisms tumors use to adapt to targeted therapies. Mutated Kras has been a target of drug development for multiple decades due to its high prevalence in several cancers. Last year, the FDA approved the first drug targeting mutant Kras. This Kras^G12C^ inhibitor is approved for patients with Kras^G12C^ advanced non‐small cell lung cancer and other Kras^G12C^ inhibitors are currently in clinical trials [6, 7, 8]. However, data released from drugs targeting Kras^G12C^, showed that tumors find ways to bypass Kras^G12C^ inhibition [7, 9]. Here we report on a novel breast cancer *Trp53^−/−^ Kras^G12D^‐inducible* mouse model that develops tumors that also adapt to Kras inhibition. We find that the tumors that are able to overcome oncogene withdrawal adopt a mesenchymal identity and reactivate the MAPK/ERK pathway. This novel immunocompetent mouse model helps us understand the mechanisms that tumors deploy to bypass Kras inhibitors and could provide a valuable resource for determining effective methods to treat patients more durably.

## 2 Materials and methods

### 2.1 Animal care and use

Mice purchased from The Jackson Laboratory (Ben Harbor, ME, USA): Trp53^f/f^ (Stock #008462), Krt14‐Cre (Stock #004782), mT/mG (Stock #007676), C57BL/6J (#000664). The *MMTV‐rtTA* and *TetO‐Kras^G12D^* mice have been described [10, 11]. All mice were backcrossed into the C57/BL6J background for at least six generations. Only female adult mice were included in the experiments. Dox was administered through dox‐impregnated food pellets (625 mg·kg^−1^, catalog #TD.01306, Envigo, Indianapolis, IN, USA). All mice used for this study were maintained at the Stanford Veterinary Service Center in accordance with the guidelines of the Administrative Panel on Laboratory Animal Care (APLAC #10868).

### 2.2 Tissue processing

Mice‐bearing tumors were euthanized and tumors were resected. The fluorescent status of tumors was confirmed under the microscope. For immunohistochemistry, small chunks were saved in 4% PFA exchanged for 70% EtOH the next day. Remainder of the tumor was mechanically dissociated and digested in DMEM/F12 (catalog #12634028, Gibco, Langley, OK, USA) with collagenase and hyaluronidase (catalog #07912, STEMCELL Technologies Inc., Vancouver, BC, Canada) and DNAse I (catalog #LS002139, Worthington, Columbus, OH, USA) for 2.5 h with gentle pipetting every 30 min. The remainder of the digestion protocol has previously been described elsewhere [12, 13]. Cells were stained for flow cytometry or resuspended with 50% Matrigel (catalog #356234, Corning, Corning, NY, USA) prior to injection into syngeneic recipient mice.

### 2.3 FACS analysis and cell sorting

For FACS analysis and sorting, tumor single cells were stained with CD45 (clone #30‐F11, Biolegend, San Diego, CA, USA), CD31 (clone #390, Biolegend), TER119 (clone #TER119, Biolegend), EpCAM (clone #G8.8, Biolegend), CD24 (clone #M1/69, Biolegend). Debris and cell doublets were excluded using side scatter and forward scatter profiles (area and width), and dead cells were excluded using DAPI (catalog #32670, Sigma, St. Louis, MO, USA). For RT qPCR or RNA‐seq, cells were directly sorted into RNAprotect (catalog #76526, Qiagen, Germantown, MD, USA). To collect DNA (either for PCR or for WES), cell pellets were frozen down at −80 °C. FACS data were analyzed in flowjo (v10, BD, Ashland, OR, USA).

### 2.4 Tumor injection and volume measurements *in vivo*

Cells were injected into the fourth abdominal fat pad by subcutaneous injection at the base of the nipple of female C57BL/6J mice. Mice were anesthetized by a constant flow of oxygen and 2% isoflurane during the procedure. Tumor size was measured by caliper. Tumor volume was calculated with the formula: 4/3 × π × (*h* × *w* ^2^)/8, wherein *h* = height and *w* = width [14]. Tumors that had increased in size on at least two consecutive occasions were considered to have reoccurred. Data was visualized in graphpad prism (v8, GraphPad Software, San Diego, CA, USA).

### 2.5 Histology: H&E, IHC, quantification

Tissue processing, embedding, and staining with hematoxylin and eosin were done by Stanford’s Comparative Medicine Animal Histology Services. Images were acquired by the BZ‐X800 fluorescent microscope (Keyence, Itasca, IL, USA). For IHC, sections were deparaffinized, dehydrated, and microwaved for 20 min at 95 °C in Sodium Citrate Buffer (10 mm Sodium Citrate, 0.05% Tween 20, pH 6.0) for antigen retrieval. Tissue sections were incubated overnight at 4 °C with primary antibodies diluted in phosphate‐buffered saline (PBS) + 5% goat serum; anti‐phospho‐ERK1/2 (catalog# 4370S, Cell Signaling Technology, Danvers, MA, USA) at a 1:100 dilution and anti‐GFP antibody (catalog# ab13970, Abcam, Cambridge, MA, USA) at a 1 : 500 dilution. Samples were subsequently washed with PBS and were incubated with secondary antibodies diluted in PBS + 5% goat serum for 1 h at RT; goat anti‐chicken Alexa Fluor 488 (catalog # A‐11039, Invitrogen, Waltham, MA, USA) and donkey anti‐rabbit Alexa Fluor 594 (catalog # R37119, Invitrogen), both diluted at 1 : 500. All the immunofluorescence sections and cells were mounted in ProLong Gold with DAPI (catalog# P36931, ThermoFisher, Waltham, MA, USA). Images were acquired with the 20× magnification objective by the LSM 710 Meta confocal microscope (Carl Zeiss, Göttingen, Germany) or by the 10× or 20× magnification objectives BZ‐X800 fluorescent microscope (Keyence). Images were processed and quantified using imagej (National Institutes of Health, Bethesda, MD, USA). 10× or 20× objective images were analyzed for the total count of positive staining and intensity. Threshold was set standardized to negative staining controls at 20%. Images were converted to 8‐bit for binary analysis of Intensity (using the Analyze ‐> Measure function). For counts of positive staining, nuclei counts were first established using the Analyze ‐> Analyze particles function on the DAPI channel. Particle count was set to diameter 1 μm < x < 10 μm to identify nuclei and rule out background staining. The Voronoi function was used on the staining channels (GFP and RFP) to isolate staining per cell and then the Analyze ‐> Measure function was applied. The value for mean intensity was calculated using this ratio and also used to establish positive counts. *N* = 3 technical replicates were considered per experiment for statistical analysis and validated in another independent biological experiment. Significance for statistical analysis was set at *P* < 0.05.

### 2.6 RT qPCR

Taking samples stored in RNAprotect, RNA was extracted using the RNeasy micro kit (catalog #74004, Qiagen), according to the manufacturer’s instructions. For reverse transcription to cDNA, the SuperScript III First Strand Synthesis kit (catalog #18080051, Invitrogen) was used, according to the manufacturer’s instructions. After 20 rounds of preamplification with Sybr Green master mix (catalog #4364346, Applied Biosystems, Waltham, MA, USA) and Sybergreen primers for *Kras* and *Kras^G12D^*, real‐time PCR was done using the 7900HT Real Time PCR system (Applied Biosystems). Data were analyzed by SDS2.4 software and GraphPad Prism (v8). All data were normalized to *Gapdh*. Sybr Green primers: *Kras* forward: GCAGGGTTGGGCCTT ACA T; *Kras* reverse: ATGCGTCGCCACATTGAAT; *Kras^G12D^* forward: CAAGGACAAGGTGTACAGTTATGTGACT; *Kras^G12D^* reverse: GGCATCTGCTCCTGCTTTTG; *Gapdh* forward: AGGTCGGTGTGAACGGATTTG *Gapdh* reverse: TGTAGACCATGTAGTTGAGGTCA [10].

### 2.7 RNA sequencing

Taking samples stored in RNAprotect, RNA was extracted using the RNeasy micro kit (catalog #74004, Qiagen), according to the manufacturer’s instructions. At least 300 ng RNA was shared for input. Library preparation, sequencing, and initial quality control were performed by Novogene (Beijing, China). Briefly, mRNA was enriched using oligo(dT) beads followed by library generation using the NEBNext^®^ UltraTM RNA Library Prep Kit for Illumina^®^ (NEB, Ipswich, MA, USA), according to the manufacturer’s instructions. To allow for sequencing different samples at the same time, adaptor sequences were added to each library. Libraries were sequenced on the NovaSeq 6000 (Illumina, San Diego, CA, USA) generating 150‐bp paired‐end reads. Per sample, 6 GB of raw data was generated.

### 2.8 RNA sequencing alignment and processing

The sequencing data were uploaded to the Galaxy web platform [15], and the public usegalaxy.org server was used to analyze the data, following the transcriptomics tutorial provided [16, 17, 18]. Briefly, sequence quality control was done multiple times using FASTQC (Babraham Institute) and MultiQC [19]. Trim Galore! (Babraham Institute) to trim the reads, HISAT2 to map reads to the mm10 reference genome [20], featureCounts to count the aligned reads [21], limma‐voom to filter out lowly expressed genes and provide a list of differentially expressed genes and normalized counts [22].

### 2.9 Gene Set Enrichment Analysis (GSEA), single sample GSEA (ssGSEA), and visualization RNA‐seq data

Patterns in gene expression were analyzed using gsea v4.0.1 [23, 24] with the Hallmark Gene sets [25]. ssgsea (v10.0.3) analysis [24, 26] was done through genepattern [27], using gene sets created by Hollern et al. [28]. Heatmap and PCA figures were created with r (4.0.0‐4.0.4) in rstudio (1.3‐1.4) using packages gplots, ggplots2, heatmap.plus, dplyr, forcats, and svglite. The Galaxy web platform [15] volcano plot tool used ggplot2, ggrepel, dplyr.

### 2.10 Whole‐exome sequencing

For tumor samples, FACS‐sorted tumor cells were used. For normal matched controls, tail DNA was used. To isolate genomic DNA (gDNA), cells/tails were digested at 65 °C overnight in 200 μL DirectPCR Lysis Reagent (catalog# 102‐T, Viagen Biotech, Los Angeles, CA, USA) mixed with 0.5 μL Proteinase K (catalog# P8107S, NEB). Lysis was inactivated at 95 °C for 10 min. gDNA was isolated using the Genomic DNA Clean & Concentrator Kit (catalog #4065D, Zymo Research, Irvine, CA, USA), according to the manufacturer’s instructions. To obtain sufficient DNA for WES, whole genome amplification was done using the REPLI‐g mini kit (catalog# 150023, Qiagen), according to the manufacturer’s instructions. At least 1 μg of gDNA was shared for input. Library preparation, sequencing, and initial quality control were performed by Novogene. Briefly, gDNA was randomly fragmented by sonication (Covaris, Woburn, MA, USA) to DNA fragments of 180–280 bp followed by library generation and exome capture using SureSelectXT Mouse All Exon (Agilent, Santa Clara, CA, USA), according to manufacturer’s instructions. Purification was done using AMPure XP (Beckman Coulter, Brea, CA, USA), according to the manufacturer’s instructions. Quantification was done with the High Sensitivity DNA assay (Agilent) on the Bioanalyzer 2100 (Agilent), according to the manufacturer’s instructions. Libraries were sequenced on the NovaSeq 6000 (Illumina) generating 150‐bp paired‐end reads. Per sample, 10 GB of raw data was generated.

### 2.11 WES alignment and processing

The sequencing data were uploaded to the Galaxy web platform [15], and we used the public server at usegalaxy.org to check the quality of the data using FASTQC (Babraham Institute) and MultiQC [19]. The remainder of the analysis was done on the Stanford Sherlock cluster. Trim Galore! (Babraham Institute) to trim the reads, reads were aligned to the mm10 reference genome with BWA‐MEM [29], duplicates were marked and removed by markduplicates (picard, gatk 4.1.4.1) [30].

### 2.12 Mutation calling and analysis

Mutect2 called mutations, FilterMutectCalls marked filtered calls, and SelectVariants filtered the output (gatk 4.1.4.1) [30]. Effects of variants were determined using ensembl’s vep v101 [31], converted to maf using vcf2maf v1.6.19 [32], and summarized using maftools v0.9.30 [33].

### 2.13 CNA analysis

Copy number analysis was performed on WES data of 10 tumor samples using sequenza 3.0.0 software package [34], including matched normal tissue to improve the specificity of results. Cellularity and ploidy values were estimated, and somatic CNAs were detected and visualized per sample by calculating the depth ratio (log2 ratio) of each segment. Thresholds for determining the CNA state for copy number gains/losses were defined as ± 0.25 and amplifications/deletions ± 1, with *P*‐value threshold being 0.05, and the results were visualized using copynumber package 1.30.0 in r [35]. Annotation of recurrent CNAs was performed with a custom script in r (4.0.4) using biomart (2.46.3) [36].

### 2.14 PCR

gDNA was isolated as for WES. PCR primers used to determine whether *Trp53* was floxed out or not: p53–int1–fwd: CACAAAAACAGGTTAAACCCA; p53–int10–fwd AAGGGGTATGAGGGACAAGG; p53–int10–rev: GAAGACAGAAAAGGGGAGGG [37].

## 3 Results

### 3.1 Generation of a *Trp53^−/−^ Kras^G12D^*‐inducible mouse model

To study the mechanisms of tumors bypassing *Kras^G12D^* inhibition *in vivo*, we used the *MMTV‐rtTA TetO‐Kras^G12D^* mouse. In *MMTV‐rtTA TetO‐Kras^G12D^* mice, the expression of *Kras^G12D^* is activated in the mammary gland in the presence of doxycycline (dox) [10, 11, 38]. Since in human breast cancer, Kras genetic alterations frequently co‐occur with *TP53* genetic alterations (Fig. S1A), we crossed the *MMTV‐rtTA TetO‐Kras^G12D^* mouse with the *Krt14‐Cre mT/mG Trp53^f/f^* (*Trp53 KO*) mouse. In the *Trp53 KO* mouse, epithelium‐specific Cre recombinase (Cre) activity triggers the deletion of *Trp53* [39, 40]. In parallel, Cre inactivates the expression of *tdTomato* and activates the expression of *GFP* in the *mT/mG* allele [41]. We have described the *Trp53 knockout* (*KO*) mouse model previously [13]. The various crosses lead to the two following mouse models which are used in this study: (a) *Trp53 KO iKras^G12D^*; *Krt14‐Cre mT/mG Trp53^f/f^ MMTV‐rtTA TetO‐Kras^G12D^* (Fig. S1B) and (b) *iKras^G12D^*; *Krt14‐Cre mT/mG MMTV‐rtTA TetO‐Kras^G12D^*. In brief, the *Trp53 KO iKras^G12^* mouse mammary gland does not express *Trp53* and oncogenic *Kras^G12D^* expression is induced in the presence of doxycycline. The *iKras^G12D^* mouse only has the inducible *Kras^G12D^* construct.

### 3.2 A subset of *Trp53^−/−^* tumors bypasses *Kras^G12D^* withdrawal and resumes proliferation

**Fig. 1:**
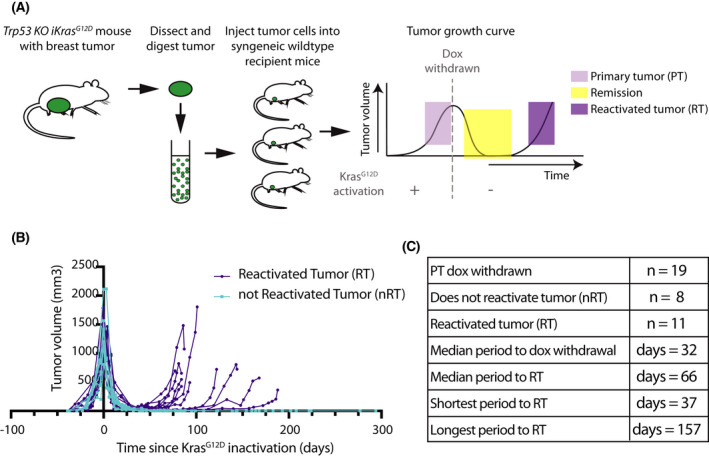
Mammary tumor formation in the *Trp53 knockout (KO) iKras^G12D^* mouse model (A) Schematic showing the *Trp53 KO iKras^G12D^* mouse model and the tumor growth curve. (B) Tumor growth curves of tumors where *Kras^G12D^* activation led to tumor growth and *Kras^G12D^* inactivation (day 0) led to either a reactivated tumor (RT, *n* = 11) or led to a not reactivated tumor (nRT, *n* = 8). (C) Table showing the data displayed in B. Median period to doxycycline (dox) withdrawal is time between the injection of tumor cells to dox withdrawal. Median period to RT is the time between dox withdrawal and a minimum of doubling of the tumor in size after remission with continued growth thereafter.

Upon the expression of *Kras^G12D^,* 25.8% (8 out of 31) of *Trp53 KO iKras^G12D^* mice developed mammary gland tumors, while none of the *iKras^G12D^* mice developed mammary gland tumors (0 out of 15) (Fig. S1C). Analyzing the expression of the epithelial cell surface marker EpCAM by flow cytometry in the 8 *Trp53 KO iKras^G12D^* tumors, we found 3 types of *Kras^G12D^* tumors: a predominantly EpCAM^high^ tumor, a predominantly EpCAM^low^ tumor, and a mixed tumor with similar proportions of EpCAM^high^ and EpCAM^low^ cells. To study the impact of *Kras^G12D^* withdrawal, we injected *Trp53 KO iKras^G12D^* tumors into syngeneic recipient mice. The EpCAM^low^ and the mixed tumor types did not dependent on the inducible Kras^G12D^ protein and continued growing upon dox withdrawal. Among the 6 predominantly EpCAM^high^ tumors, 4 established new tumors in syngeneic mice of which 2 also did not dependent on *Kras^G12D^* expression. This study focuses on the 2 remaining EpCAM^high^ tumors: tumors that declined rapidly upon dox withdrawal, thereby showing to initially dependent on *Kras^G12D^* expression for tumor formation (Primary Tumor; PT) (Fig. 1A, Fig. S1C). After a median period of 66 days, nearly 60% (11 out of 19) of the tumors in remission had adapted to the absence of Kras^G12D^ and reactivated tumor growth (Reactivated Tumor; RT) (Fig. 1B,C). We confirmed transgenic *Kras^G12D^* expression was not restored in RTs (Fig. S2A) and the successful excision of *Trp53* by PCR in all tumors was confirmed (Fig. S2B). The PTs and RTs formed in the *Trp53 KO iKras^G12D^* mouse model enabled us to study how mammary gland tumors overcome the withdrawal of oncogene expression. Taken together, these results show that tumors adapt to Kras^G12D^ inactivation.

### 3.3 Reactivated tumors are enriched for EMT markers

**Fig. 2:**
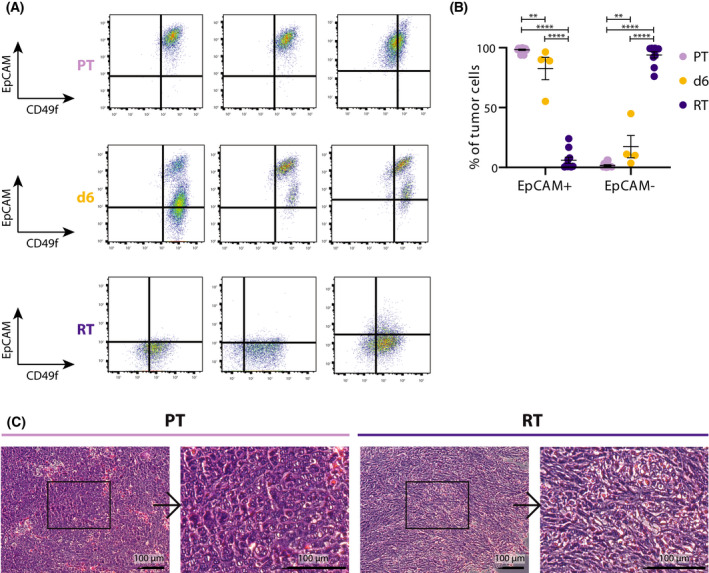
The RT loses EpCAM expression upon *Kras^G12D^* inactivation and becomes mesenchymal‐like (A) Representative FACS plots of PT, RT, and 6 days after dox withdrawal (d6) tumors. EpCAM and CD49f expression of fluorescent tumor cells, lineage (Ter119/CD31/CD45)‐DAPI. PT *n* = 3, d6 *n* = 3, RT *n* = 3. (B) The percentage of EpCAM^high^ and EpCAM^low^ cells in the PT, d6, and RT tumors. PT *n* = 14, d6 *n* = 4, RT *n* = 10. Mean ± SEM is shown (2‐way ANOVA, **P < 0.005, ****P < 0.0001). (C) Pathology, H&E staining. PTs have an epithelial, invasive phenotype. RT have a stromal, spindle‐like, mesenchymal‐like, invasive phenotype. Scale bar is set at 100 μm.

**Fig. 3:**
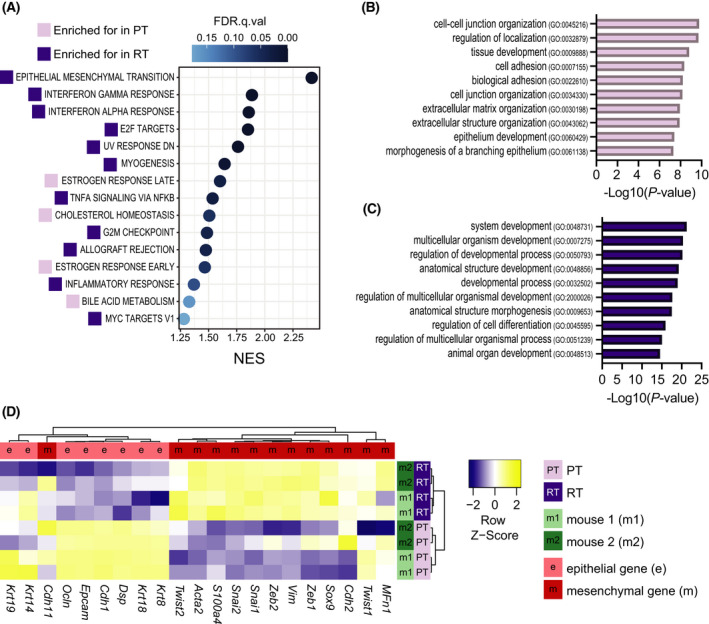
RTs display a strong (epithelial‐mesenchymal transition) EMT profile. Comparison of the transcriptomes of 4 PTs and 4 RTs. (A) Gene Set Enrichment Analysis (GSEA) hallmark gene sets upregulated in PTs and RTs (nom. *P*‐value < 0.05, FDR *q*‐value < 0.25). (B) Top 10 gene ontology terms enriched in PT. (C) Top 10 gene ontology terms enriched in RT. (D) Heat map of the expression of epithelial and mesenchymal marker genes.

To learn more about the PT and the RT, we analyzed EpCAM and CD49f expression on both tumor types and on tumors 6 days after dox withdrawal, using flow cytometry. In the absence of dox, there was a rapid reduction in the number of EpCAM^high^ cells. Once the tumor resumed to grow again, the tumor consisted predominantly of EpCAM^low^ tumor cells (Fig. 2A,B). The change in EpCAM expression between *Trp53 KO iKras^G12D^* primary and reactivated tumors, suggested a switch in tumor type since EpCAM is an epithelial cell marker usually absent in mesenchymal tumors. In support, H&E staining showed changes in tumor histology: PTs displayed an epithelial, invasive phenotype and RTs a stromal, spindle‐like, mesenchymal‐like, invasive phenotype (Fig. 2C, Fig. S2C). To study the differences between PTs and RTs in more detail, we performed RNA‐seq (Fig. S3A,B). Gene set enrichment analysis (GSEA) displayed multiple differences between the two tumor types. The strongest enrichment was found in the epithelial‐mesenchymal transition (EMT) hallmark gene set in RTs (Fig. 3A). Moreover, the gene ontology annotations enriched were cell adhesion and extracellular organization in PTs (Fig. 3B) and developmental processes in RTs (Fig. 3C). In addition, multiple epithelial genes clustered in the PTs and mesenchymal genes in the RTs (Fig. 3D). Lastly, a single sample GSEA (ssGSEA) with a signature set developed to distinguish different tumor types in mice [28], confirmed that the PTs expressed an EMT down signature while the RTs expressed an EMT up signature (Fig. S3C). Together, flow cytometry, histology, and RNA‐Seq data showed a change from a dominant epithelial phenotype in the PT to a dominant mesenchymal phenotype in the RT.

### 3.4 Reactivated tumors display active MAPK/ERK signaling

**Fig. 4:**
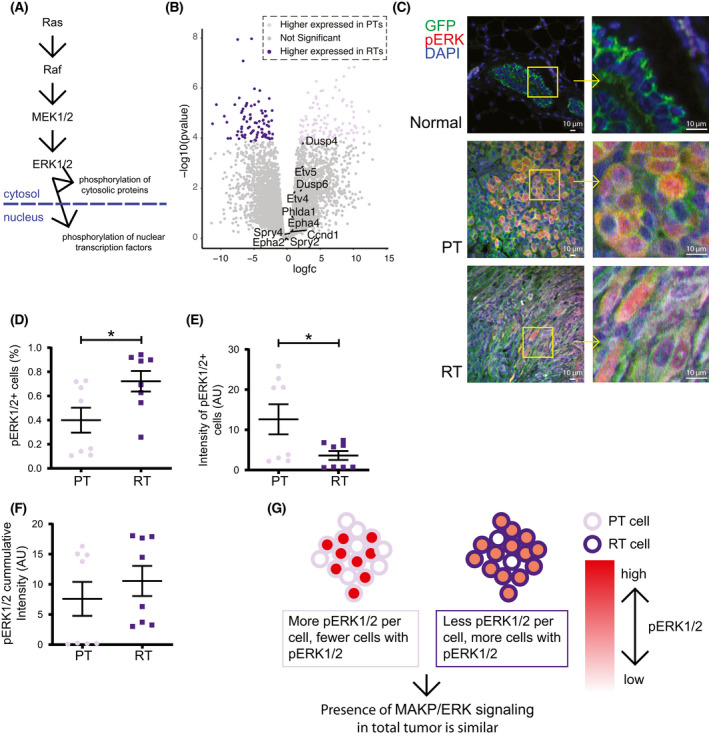
RTs activate RAS‐RAF‐MEK‐ERK (MAPK/ERK) signaling (A) Simplified schematic of the MAPK/ERK phosphorylation signal transduction cascade. (B) Volcano plot displaying the transcriptome highlighting differential expressed genes in color (FDR < 0.01, fold change > 1.5). MAPK Pathway Activity Score genes [42] are indicated. PT *n* = 4, RT *n* = 4. (C) Immunofluorescence staining for phosphorylated ERK1/2 (pERK1/2) (red), GFP (green), and DAPI (blue) of tissue deriving from a normal *Krt14‐Cre mT/mG* mammary gland, a PT, and an RT. Scale bar is set at 10 μm. Images of a biological replicate are shown in Fig S5. (D) The percentage of cells expressing pERK1/2 in PTs and RTs. Quantification of each 4 images of 2 biological controls of PTs and RTs is shown. Data are represented as mean ± SEM (unpaired *t* test, *P < 0.05). (E) The intensity of pERK1/2 staining in pERK1/2‐positive cells. Quantification of each 4 images of 2 biological controls of PTs and RTs are shown. Data are represented as mean ± SEM. (unpaired *t* test, *P < 0.05) (F) The cumulative intensity of pERK1/2 staining in the tumors. Quantification of each 4 images of 2 biological controls of PTs and RTs are shown. Data are represented as mean ± SEM. (unpaired *t* test, n.s.) (G) Model: constitutive active Kras^G12D^ results in strong MAPK/ERK signaling in a subset of the PT cells. RT cells do not reach as strong of a phosphorylation cascade per cell as in some PT cells. However, since a large percentage of individual RT cells activate MAPK/ERK signaling, the result for both tumor types as a whole is similar MAPK/ERK signaling.

Since the GSEA analysis showed no enrichment of *Kras* signaling in PTs, we looked deeper into the activity of the MAPK/ERK pathway in both tumor types. The MAPK/ERK signaling cascade commences when a phosphorylated receptor tyrosine kinase (RTK) catalyzes the activation of GTPase Ras. Subsequently, this leads to a catalyzation cascade of related protein‐serine/threonine kinases ultimately steering transcription in the nucleus (Fig. 4A). Focusing on a group of genes known to give accurate predictions of MAPK/ERK pathway activity [42], we found no change in the activity of the MAPK/ERK pathway in RTs (Fig. 4B). To elucidate this observation, we stained for phosphorylated ERK1/2 (pERK1/2), a marker of activated MAPK/ERK signaling. In the normal adult mammary gland, the vast majority of mammary epithelial cells did not express pERK1/2. Unlike PT and the RT cancer cells, where pERK1/2 was abundant (Fig. 4C, Fig. S4). Quantification showed that the RTs contained a higher number (Fig. 4D) but lower per cell presence of pERK1/2 (Fig. 4E). Cumulatively, similar amounts of pERK1/2 were present in the PTs and RTs (Fig. 4F). Together, this data showed that in the process of bypassing the loss of *Kras^G12D^* expression, RT cells are either able to maintain and/or reactivate MAPK/ERK signaling (Fig. 4G).

### 3.5 The development of tumor reactivation cannot be explained by mutational data

**Fig. 5:**
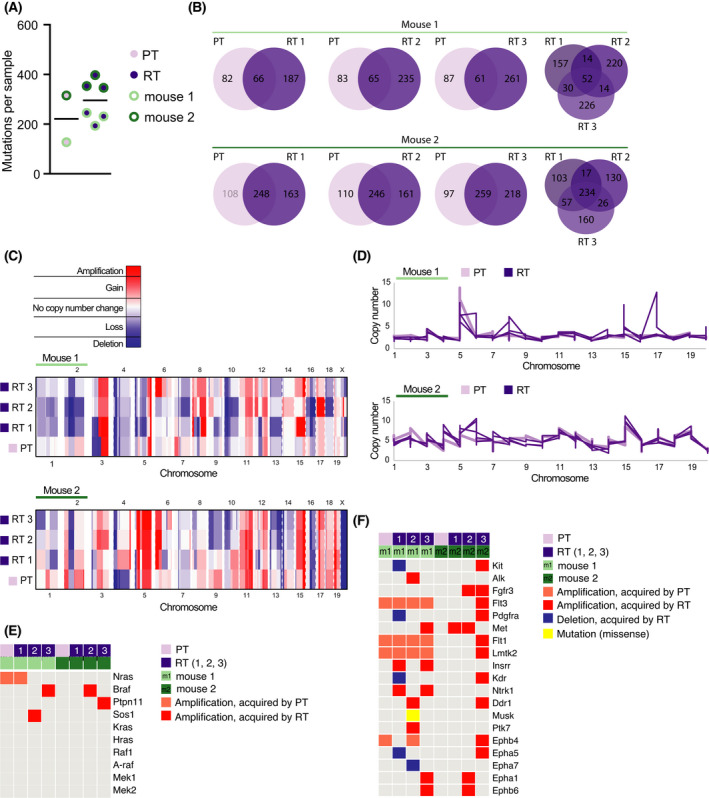
Tumors are genetically unstable and acquire multiple alterations that can activate MAPK/ERK signaling (A) Number of total acquired mutations by each PT and RT. The black horizontal line shows the median. (B) Venn diagrams showing the overlap and divergence of mutations acquired by a PT and its matched RTs (RT 1, 2, 3) and the overlap between mutations acquired by RTs originating from the same PT (mouse 1, mouse 2) (Venn diagram tool: [43]). (C) Copy number profiles with thresholds of ± 0.25 for gains/losses and ± 1 for amplifications/deletions are shown for PT and matched RTs compared with their matched normal genome. Heatmaps show PTs and their 3 matched RTs (RT 1, 2, 3) in both mice (mouse 1, mouse 2) separately. Red indicates gain/amplified and blue loss/deleted regions. (D) Absolute copy number profiles genome‐wide for PTs and the 3 matched RTs are shown. Absolute copy numbers are computed based on logR values derived from genomic segments using Sequenza. The profiles are shown separately for the 2 mice (mouse 1, mouse 2). (E) Oncoplot showing genes directly involved with MAPK/ERK signaling, 4 out of 11 have genetic alterations. RTs (RT 1, 2, 3) are shown directly next to their matched PTs. (F) Oncoplot showing receptor tyrosine kinase (RTK) genes with genetic alterations. RTs (RT 1, 2, 3) are shown directly next to their matched PTs.

Next, we investigated whether genetic changes are responsible for the observed MAPK/ERK signaling in RTs. Therefore, we performed whole‐exome sequencing (WES) on two PTs with each three matched RTs. All tumor samples acquired multiple mutations that continued to accumulate in the RTs (Fig. 5A, Table S1). Mutations were mostly single nucleotide variants (SNVs) (Fig. S5A). The nonsynonymous mutations were mostly missense mutations (Fig. S5B). Comparing the mutational profiles of the matched tumors, not all mutations found in the PT were present in the matched RTs and RTs showed different mutations among themselves (Fig. 5B). This suggested that each PT was a heterogeneous tumor with multiple clones that acquired different mutations. Next, aiming to identify mutations that could be responsible for the *Kras^G12D^*‐independent activation of MAPK/ERK signaling, we looked for overlap in genes with nonsynonymous mutations in various samples (Fig. S5C). Specifically, we selected altered genes that were present in at least one RT of each mouse and absent in either PT. However, the 7 genes matching this description (*Clasrp, Cyp3a44, Gpr18, Lrp2, Map1b*, *Spen*, and *Ttn*), are neither known breast cancer or pan‐cancer driver genes nor directly involved with the activation of MAPK/ERK signaling (Fig. S5D, Table S2). Of note, the two PTs did not have any mutated genes in common, therefore acquired mutations by the PTs do not explain why these two mice developed breast cancer in contrast to the majority of *Tp53 KO iKras^G12D^* mice. Using our dataset, nonsynonymous mutations did not play a clear role in tumor adaptation.

### 3.6 Various MAPK/ERK pathway genes are amplified in reactivated tumors

Following the analysis of the tumors’ mutational profiles, we looked at the copy number alteration (CNA) landscape of the PTs and matched RTs (Table S3). CNA data showed a high level of chromosome instability (Fig. 5C, Fig. S6). The widespread increase in copy number was already present in PTs (Fig. 5D). On an individual sample level, we looked for amplified or deleted genes that are either part of the MAPK/ERK signaling pathway or could activate the MAPK/ERK signaling pathway, such as RTKs. Many of these genes were amplified in one or multiple RTs while showing no copy number alterations in their matched PTs and no deletions in any of the other samples (Fig. 5E,F). Hence, the amplification of various components of the MAPK/ERK signaling pathway could provide a mechanism for how tumors restore or maintain active MAPK/ERK signaling.

## 4 Discussion

After decades of research, the FDA approved the first Kras inhibitor last year, targeting oncogenic Kras^G12C^. Although a huge milestone, many tumors use mechanisms to adapt to Kras^G12C^ inhibition, resulting in only temporary relief for patients [7, 9]. To study what mechanisms tumors deploy to adapt to *Kras* inactivation, we developed the *Trp53 KO iKras^G12D^* mouse model. The *Trp53 KO iKras^G12D^* mouse model spontaneously presented with tumors that have found methods to bypass *Kras^G12D^* inactivation after a period of remission. Since C57BL/6J *iKras* mice did not develop mammary gland tumors, the absence of *Trp53* in the *Trp53 KO iKras* mice played a role in the enablement of tumorigenesis. Previous research showed that it is context‐specific if the sole expression of oncogenic *Ras* is sufficient to induce tumorigenesis [44, 45, 46, 47, 48]. The absence of tumor formation is attributed to induced senescence in cells expressing high levels of oncogenic *Ras*, regulated by tumor suppressor genes such as *Trp53* and *Cdkn2a* [44, 47]. In a mammary gland *Cdkn2a KO* mouse model, the expression of *Hras^G12V^* resulted in bigger tumors in *Cdkn2a KO* tumors than in *Cdkn2a wildtype* tumors [47]. In contrast to our data, a recent study found that the sole expression of *Kras^G12D^* is sufficient to induce mammary tumor formation in the mouse mammary gland. Using an *MMTV‐tTA TetO‐Kras^G12D^* mouse, Rädler et al. [46] observed mammary tumor formation in 100% of mice with an average latency of 160 ± 41 days. There are some important differences between the two studies. We used the *Tet‐On* system, activating the expression of *Kras^G12D^* in adult mice, while Rädler et al. used the *Tet‐Off* system, where *Kras^G12D^* is expressed continuously until actively turned off. In addition, the two studies worked with different mouse strains. We backcrossed our mice into the C57BL/6J background, Rädler et al. created their mouse in the FVB/N genetic background, a commonly used strain in cancer research and for the creation of novel transgenic models. We chose the C57BL/6 mouse strain due to its low susceptibility to spontaneous tumor formation [49, 50, 51, 52, 53], which is a likely explanation for why only a minority of *Trp53 KO iKras* and none of the *iKras* mice developed mammary tumors. This is different in the FVB/N strain, at 14 months of age 26% of female wildtype FVB/N mice were tumor‐bearing [51]. A direct comparison between FVB/N and C57BL/6J breast tumor mouse models has been done with the use of the PyMT transgene, a popular transgenic breast cancer mouse model due to a short latency in primary mammary tumor development and metastasis to the lungs [54]. When FVB/N‐PyMT are crossed with only one generation of C57BL/6J mice, the latency of primary and metastatic tumor development stretches out significantly [50], and the latency period further increases when PyMT is backcrossed for at least five generations of C57BL/6J mice [55]. Other benefits of using the C57BL/6J strain are the availability of high‐quality genomic data [56] and the absence of an immune response that could clear GFP or tdTomato expressing cells [57, 58, 59, 60, 61, 62]. Low susceptibility to spontaneous tumor formation, the availability of genomic reference data, and the tolerability of fluorescent proteins were important reasons for us to choose the C57BL/6J strain.

In our *Trp53 KO iKras* model, we observed that 58% of tumors reoccur. These tumors showed a strong EMT signature that is absent in the PTs. Other studies have shown a connection between EMT and resistance to RAS inhibition using cell lines. In human breast immortalized cell lines, EMT‐marker *ZEB1* overcame *HRAS* induced senescence [63]. Lung and pancreatic cancer cell lines that depend on Kras expression were uniformly epithelial while Kras‐independent cell lines were not [64]. Kras^G12C^‐mutant lung cancer cell lines that displayed both intrinsic and acquired resistance to a Kras^G12C^ inhibitor had undergone EMT [65]. In addition, EMT is thought to play an important role in tumor cell plasticity. Reports showed that the transition from an epithelial to a mesenchymal tumor can stimulate chemoresistance [66, 67]. In the HER2/neu‐inducible breast cancer mouse model, recurrent tumors displayed an EMT signature [68] and an EMT shift has been observed in a portion of lung cancer patient samples that acquired resistance to EGFR inhibitors [69]. Together, our data and published studies suggest that tumor cell plasticity—changing a cell’s phenotype from an epithelial to a mesenchymal phenotype—fuels tumor cells with a mechanism to escape suppression [70].

In accordance with what we observed in our mouse model, activating mutations and amplification of MAPK/ERK signaling are also observed in patients that acquired resistance to Kras^G12C^ inhibitors [71, 72]. Although a direct correlation between the increased copy numbers of multiple upstream and downstream players of *Kras* and the continuation or reactivation of MAPK/ERK signaling we observed, still needs to be shown. Furthermore, upregulated pERK has also been detected in colorectal Kras^G12C^‐mutant cancer cell lines treated with Kras^G12C^ inhibitors, after a short period of downregulated pERK [73]. The Kras^G12C^ inhibitors currently tested or approved for patients, represent the first generation of Kras inhibitors. Next generation inhibitors are already being developed. For example, drugs that can inhibit the active GTP‐bound form of Kras^G12C^ [72, 74, 75]. Furthermore, combination therapy targeting other ERK/MAPK players simultaneously could benefit patients. Preclinical data have shown that combining a Kras^G12C^ inhibitor with an EGFR antibody or an SHP2 inhibitor – two proteins active upstream of RAS – diminished tumor growth in Kras^G12C^‐mutant cancers [73, 76].

In breast cancer, 13% of endocrine‐resistant advanced breast cancer develop genetic alterations in MAPK/ERK pathway genes, including Kras [77]. Our data showed that also *Kras^G12D^*‐independent breast tumors displayed active MAPK/ERK signaling, this would be in line with the importance of the MAPK/ERK pathway for tumor growth. The phenomenon of tumors with active MAPK/ERK signaling as a mechanism of adaptation has been reported for other MAPK/ERK cascade players too. For example, colon cancer patients with the BRAF^V600E^ mutation showed limited response to BRAF^V600E^ inhibitors. BRAF^V600E^ mutated colorectal cell lines showed that feedback mechanisms upregulate upstream EGFR signaling [78, 79]. Another mechanism of adaptation is found in defiance of MEK1/2 inhibitors, inhibitor‐treated colorectal, and lung cancer cell lines displayed intrachromosomal amplification of mutant Kras or BRAF or upregulation of upstream RTK signaling [80, 81]. In case of Kras^G12C^ inhibitors, Kras*^G12C^* mutated lung cancer cell lines initially entered quiescence. However, a subset of tumor cells adapted and quickly resumes proliferation [82]. In mutant Kras pancreatic ductal adenocarcinoma cell lines, Kras inhibition was well tolerated by the tumor [83]. Kras^G12C^ drug‐resistant cells displayed both active MAPK/ERK signaling and active PI3K‐AKT–mTOR signaling, a second pathway that can be activated by Kras [65, 84]. These examples suggest that targeting multiple components of the MAPK/ERK pathway may provide a strategy for eliminating or at least delaying tumor adaptation. In the clinic, this strategy has already proven to be successful for some patients with BRAF^V600^‐mutant metastatic melanoma as therapy combining a MEK1/2 inhibitor with a BRAF^V600E^ inhibitor was successfully applied [85]. We endeavored to test whether inhibiting multiple MAPK/ERK players simultaneously can prevent RTs from growing *in vitro*. Unfortunately, the RT cells did not form organoids *in vitro*, hence we did not succeed in testing this hypothesis. Perhaps this is due to the mesenchymal phenotype of RTs, colorectal cancer samples with a mesenchymal phenotype fail to form organoids as well [86].

Another observation was the high genomic instability of the PTs and RTs. Genomic instability is an important hallmark of cancer. In human cancers, genomic instability occurs in 88% of tumors and correlates with *TP53* mutations [87]. In breast cancer, aneuploidy is correlated with poor clinical outcomes and *TP53* mutations are ubiquitous in aneuploid breast tumors [88, 89, 90]. Breast cancer mouse models that do not directly act through p53 inhibition, displayed few or no CNAs [91]. In addition, *Kras^G12D^* lung and pancreas tumor mouse models also developed widespread CNAs [92]. Thus, mouse models ‐ such as the *Trp53 KO iKras^G12D^* model described here – that develop spontaneous tumors with CNAs provide an important model for human cancer. In line with other *Trp53* deficient breast cancer models [91], both the *Trp53 KO iKras^G12D^* primary and reactivated tumors presented with high rates of genomic instability.

## 5 Conclusions

The present study aimed to understand what mechanisms tumor cells deploy to adapt to mutant Kras inactivation *in vivo*. In our novel breast cancer mouse model, we found that once *Kras^G12D^* expression was inactivated, tumors decreased and entered remission, followed by reactivated tumor growth. RT cells had transitioned from an epithelial to a mesenchymal phenotype and displayed active MAPK/ERK signaling. Tumors presented with high genomic instability and RTs showed amplification in multiple genes associated with MAPK/ERK signaling. Our data suggest that the genomic instability contributes to the emergence of mutated Kras‐independence via amplification of various components of the MAPK/ERK signaling pathway. These findings are relevant to therapeutics targeting RAS in human clinical trials.

## Conflict of interest

The authors declare no conflict of interest.

## Author contributions

LJvW, SC, FAS, and MFC conceived the study and designed the experiments. LJvW, SID, and MFC wrote the manuscript. LJvW performed and analyzed most of the experiments. SID analyzed the CNA data. SSS performed the fluorescence IHC. JA quantified the fluorescence IHC images. DQ and WHDH aided with mouse genotyping and tissue processing. RW analyzed the H&E histology data.

## Supporting information


**Fig. S1.** Tumor formation in *Trp53 KO iKras^G12D^* mice (A) Oncoplot of human breast cancer samples showing genetic alterations in the *Kras* and *TP53* genes, n = 4925. Only patient samples with genetic alterations in at least one of these two genes are shown. Data are compiled from 3 studies [77, 95, 96, 97] (Co‐occurrence genetic alterations in both genes q‐value = < 0.001). (B) The various components of the mouse model. In cell expression *Krt14*, such as all mammary gland epithelial cells during development, *Cre recombinase* (*Cre*) is expressed. Cre deletes part of the *Trp53* gene, thereby eliminating the expression of *Trp53*. Cre also deletes the gene coding for membrane‐bound *tdTomato* and a STOP codon inhibiting membrane‐bound *GFP* expression, resulting in the expression of membrane‐bound GFP. The MMTV promoter is expressed in the mammary epithelium. rtTA can only bind to the *TetO* promoter in the presence of dox, resulting in the expression of *Kras^G12D^*. Therefore, in the absence of dox, *Kras^G12D^* is not expressed. (C) Tumor frequency and survival plots of all *Trp53 KO iKras^G12D^* and *iKras^G12D^* mice in this study. FACS profiles and tumor growth profiles upon transgenic *Kras^G12D^* activation and inactivation by dox in *Trp53 KO iKras^G12D^* mice that developed mammary gland tumors. The focus of this study are tumors that initially go into remission upon *Kras^G12D^* inactivation, followed by *Kras^G12D^*‐independent reactivation.

**Fig. S2.** Verification of the *Trp53 KO iKras^G12D^* mouse model (A) RT qPCR data showing the expression of *Kras* and *Kras^G12D^* in PTs (n = 5) and in RTs (n = 4). Data are represented as mean ± SEM (unpaired t test, **P < 0.01). (B) PCR data confirming the presence (*Trp53* wildtype) or absence (*Trp53 KO*) of the Trp53 allele in *Trp53 KO iKras^G12D^* tumor cells, control is wildtype tail DNA. (C) Additional H&E staining from PT and RT tumors, in addition to slides shown in Fig. 2C. Scale bar is set at 100 mμ.

**Fig. S3.** RT and PT samples cluster and RNA‐seq confirms EMT profile in RT tumors (A) PCA of PT (4) and RT (4) RNA‐seq samples. (B) Heatmap of RNA‐seq expression profiles showing all filtered and normalized counts. (C) A single sample GSEA (ssGSEA) gene set developed to analyze mouse tumor histology. Gene sets for EMT enriched and EMT decreased are shown. Gene sets were developed by Hollern et al. [28].

**Fig. S4.** RTs activates MAPK/ERK signaling, additional data Figure in addition to Fig. 5C, showing a second set of biological samples in an experiment performed separately, using a different fluorescent microscope for imaging. Immunofluorescence staining for pERK1/2 (red), GFP (green), and DAPI (blue) of tissue deriving from a normal *Krt14‐Cre mT/mG* mammary gland, a PT, and an RT. Scale bar is set at 100 mμ.

**Fig. S5.** Analysis mutational data (A) Number of single nucleotide variants (SNVs) and INDELs acquired by each PT and RT. The black horizontal line shows the median. (B) Pie chart showing the average percentage of nonsynonymous and synonymous mutations in PTs and RTs and the average percentage of the different types of nonsynonymous mutations in PTs and RTs. (C) Venn diagram of overlap between genes with nonsynonymous mutations in PT and 3 matched RTs (RT1‐RT3), mouse 1 and mouse 2 (Venn diagram tool: [43]). (D) Overview of genes with nonsynonymous mutations in at least 1 RT (RT1/RT2/RT3) of each mouse (m1/m2) and no mutations in either PT. The resulting 7 genes were compared with MAPK/ERK genes, RTK genes, pan cancer, and breast cancer driver genes [1, 4, 98].

**Fig. S6.** Allele‐specific copy number analysis for 2 PTs and 6 RTs against their matched normal counterparts Analysis was performed using the Sequenza algorithm for paired tumor‐normal whole‐exome sequencing (WES) data to visualize copy number alterations (CNAs) in each sample as described by raw copy number, depth ratio (logR), and b‐allele frequency profiles. Red and blue represent the overall copy number and minor allele, respectively.

**Table S1.** Overview mutations.

**Table S2.** Overview of genes with nonsynonymous mutations and information on their human homolog pan and breast cancer driver status.

**Table S3.** Unannotated CNA data.

## Data Availability

Human breast cancer data were accessed through cBioportal.org [93, 94] on 27 April 2021 and included data with both mutational and CNA data (n = 5087) from the TCGA PanCancer atlas at www.cancer.gov/tcga [95], the METABRIC study at http://doi.org/10.1016/j.ccell.2018.08.008 and http://doi.org/10.1038/nature10983 [96, 97], and MSKCC data at http://doi.org/10.1016/j.ccell.2018.08.008 [77]. RNA‐seq data that support the findings in this study are openly available in the Gene Expression Omnibus at NCBI (www.ncbi.nlm.nih.gov/geo/) under accession number GSE174441. The nucleotide sequence data that support the findings in this study are openly available in the Sequence Read Archive at NCBI (www.ncbi.nlm.nih.gov/sra) under accession number PRJNA730907.
